# Hydatid cyst of the fallopian tube; Case report with literature review

**DOI:** 10.1016/j.ijscr.2019.11.048

**Published:** 2019-12-03

**Authors:** Dildar Haji Musa, Ayad Ahmad Mohammed

**Affiliations:** Department of Surgery, College of Medicine, University of Duhok, Kurdistan Region, Iraq

**Keywords:** Hydatid disease, Echinococcus, Fallopian tube, Adnexial cyst, Ovarian cyst, Salpingectomy

## Abstract

**Introduction:**

Hydatid disease is a zoonotic disease which is caused by a parasite belonging to the Echinococcus species. It may affect all part of the body mostly the liver and the lungs, involvement of the fallopian tube is a very rare.

**Case presentation:**

A 30-year-female presented with acute lower abdominal pain, fever and vomiting for 2 days. The past medical history was negative and she had history of surgery for hydatid cyst of the right lobe of liver 3 years before presentation. During examination there was tenderness at the right iliac fossa with guarding. Ultrasound of the pelvic organs showed a left adnexial cyst. During surgery there was an evidence of hydatid cyst involving the left fallopian tube. Left salpingectomy was performed with excision of the cyst. The patient remained three days in the hospital with no reported complication.

**Conclusion:**

Hydatid cyst is one of the differential diagnoses of any cystic lesion in patients from an endemic areas of hydatid disease. Hydatid cysts of the female pelvic organs may be diagnosed initially as ovarian cyst, ovarian tumor, or pelvic infection. Most of the cases are diagnosed during surgery which is the main treatment modality; anthelminthic medications may help to decrease the recurrence rate.

## Highlights

• Hydatid disease rarely affect the female genital organs.
• Most cases are seen in the liver and the lungs.
• Surgery is the main form of treatment which can be done laparoscopically.

## 1 Introduction

There are many zoonotic diseases that are transmitted to human beings, hydatid disease is one of the commonest types of them. This disease is caused by a parasite belonging to the Echinococcus species. The route of the transmission of this disease is through the fecal-oral route when the eggs are ingested with the contaminated food or water and reach the bowel of the accidental intermediate host; i.e. the human. The eggs are hatched and transmitted by the blood circulation to all parts of the body, hepatic and pulmonary involvements are the commonest, other organs may be affected with variable frequencies, hydatid cyst of the fallopian tube is a rare finding and in most case it is associated with involvement of other body organs [1,2].

Most cases are diagnosed incidentally or the disease may cause nonspecific symptoms. Pain is the most frequent symptom; large cysts may cause pressure symptoms, or the cyst may present with complications such as infection and rupture [3].

Imaging may be useful in making a correct preoperative diagnosis especially when the cyst contains the floating membrane or when contains many daughter cysts, it may also be helpful in delineating the anatomy and showing the involvement of other organs [2].

The work of this report case has been reported in line with the SCARE 2018 criteria [4].

## 2 Patient information

A 30-year-female presented with acute lower abdominal pain, fever and vomiting for 2 days. The past medical history was negative and the patient had history of surgery for hydatid cyst of the right lobe of liver 3 years before presentation, no spillage was reported during the first surgery, the patient received postoperative anthelminthic drugs for 3 months after the first surgery.

### 2.1 Clinical findings

There pulse rate was 110 beats /minutes, the blood pressure was 105/65 mmHg, and the temperature was 38.6 degrees of Celsius.

During abdominal examination, there was tenderness at the right iliac fossa with guarding.

### 2.2 Diagnostic assessment

Ultrasound of the pelvic organs showed a left adnexial cystic lesion measuring 60*50 mm with clear contents.

### 2.3 Therapeutic intervention

**Fig. 1:**
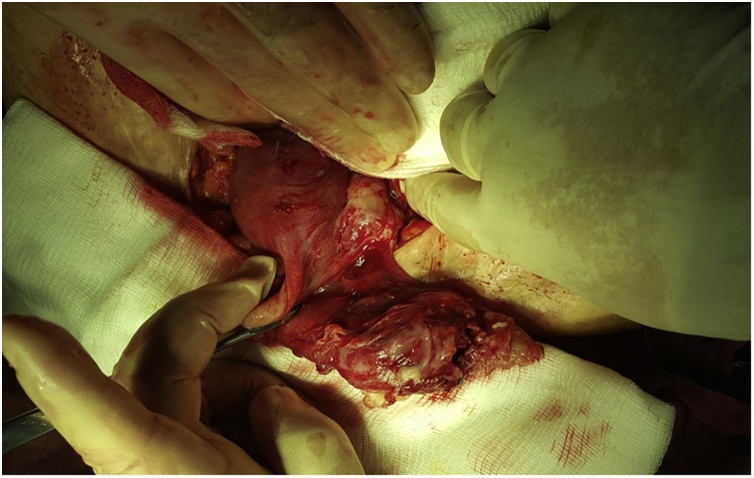
An intraoperative picture showing the hydatid cyst affecting the left fallopian tube.

Diagnostic laparoscopy was performed which showed an evidence of inflammatory mass in the left adnexial region, the surgery was then converted to the open one. There was an evidence of hydatid cyst involving the left fallopian tube Fig. 1.

**Fig. 2:**
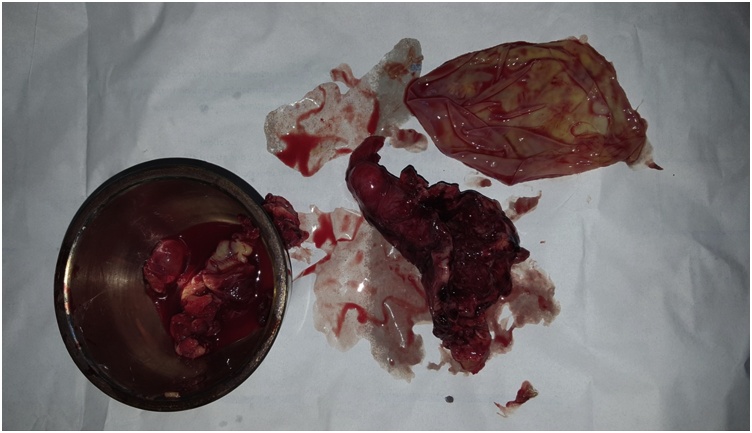
An intraoperative picture showing the fallopian tube and the hydatid cyst after successful surgical excision.

Left salpingectomy was performed with excision of the cyst, the surgical field was isolated with surgical sponges soaked with chlorhexidine solution to control any intraoperative spillage Fig. 2.

### 2.4 Follow-up and outcomes

The patient remained three days in the hospital with no reported complication, she received anthelminthic drugs for 3 months after the surgery.

## 3 Discussion

Hydatid cyst is one of the differential diagnoses of any cystic lesion in any part of the body in patients from an endemic areas of hydatid disease such as the Mediterranean region. Once hydatid cyst is diagnosed, other organ involvement should be excluded particularly pulmonary involvement as it has a priority for management before any surgical intervention for other anatomical sites [3,5].

Hydatid cysts involving the female pelvic organs may be diagnosed initially as simple or complicated ovarian cysts, dermoid cyst, ovarian tumor, or pelvic infections and abscesses. Most of the cases are diagnosed during surgery [2,6].

The blood tests are normal in the majority of cases, however, in cases of infected cysts the white blood cells may be elevated, the eosinophil count may be elevated in some cases as an allergic manifestation to the parasite [7].

In the review of literature only few cases of primary involvement of the female pelvic organs are reported. Involvement of the fallopian tubes is very rarely reported, and less than 30 cases are reported. Such cases are usually diagnosed as para-ovarian cysts and most cases are diagnosed during surgery [2,8].

Surgery is the main treatment modality, but medical treatment with anthelminthic medications such as albendazole may be effective in some occasions, the main benefit of the medical therapy is in the form of adjuvant therapy after surgery to decrease the recurrence rate. During surgery the most important step is preventing spillage of the content of the cyst to avoid recurrence, and some patients may develop severe allergic reaction to the cyst fluid, cases of intraoperative deaths have been reported. Follow up is recommended to detect early recurrence and to detect other organs involvement [7,9].

## Funding

None.

## Ethical approval

Ethical approval has been exempted by my institution for reporting this case.

## Consent

Written informed consent was obtained from the patient for publication of this case report and accompanying images.

## Author contribution

The concept of reporting the case, data recording, and drafting the work done by Dr Dildar Haji Musa and Dr Ayad Ahmad Mohammed

Dr Dildar Haji Musa took the consent from the patient for publishing the case.

Final approval of the work to be published was done by Dr Ayad Ahmad Mohammed.

## Registration of research studies

This work is case report and there is no need of registration

## Guarantor

Dr Ayad Ahmad Mohammed is guarantor for the work.

## Provenance and peer review

Not commissioned, externally peer-reviewed.

## Patient’s perspective

After surgery I am afraid of recurrence of the cyst, I should keep regular checks with my doctor.

## Declaration of Competing Interest

The author has no conflicts of interest to declare
